# Single cell analysis of the inner ear sensory organs

**DOI:** 10.1387/ijdb.160453ka

**Published:** 2017

**Authors:** OFER YIZHAR-BARNEA, KAREN B. AVRAHAM

**Affiliations:** Department of Human Molecular Genetics and Biochemistry, Sackler Faculty of Medicine and Sagol School of Neuroscience, Tel Aviv University, Tel Aviv, Israel

**Keywords:** cochlea, vestibule, mice, hearing, deafness, transcriptome

## Abstract

The inner ear is composed of a complex mixture of cells, which together allow organisms to hear and maintain balance. The cells in the inner ear, which undergo an extraordinary process of development, have only recently begun to be studied on an individual level. As it has recently become clear that individual cells, previously considered to be of uniform character, may differ dramatically from each other, the need to study cell-to-cell variation, along with distinct transcriptional and regulatory signatures, has taken hold in the scientific community. In conjunction with high-throughput technologies, attempts are underway to dissect the inter- and intra-cellular variability of different cell types and developmental states of the inner ear from a novel perspective. Single cell analysis of the inner ear sensory organs holds the promise of providing a significant boost in building an *omics* network that translates into a comprehensive understanding of the mechanisms of hearing and balance. These networks may uncover critical elements for trans-differentiation, regeneration and/or reprogramming, providing entry points for therapeutics of deafness and vestibular pathologies.

## Introduction

**Fig. 1:**
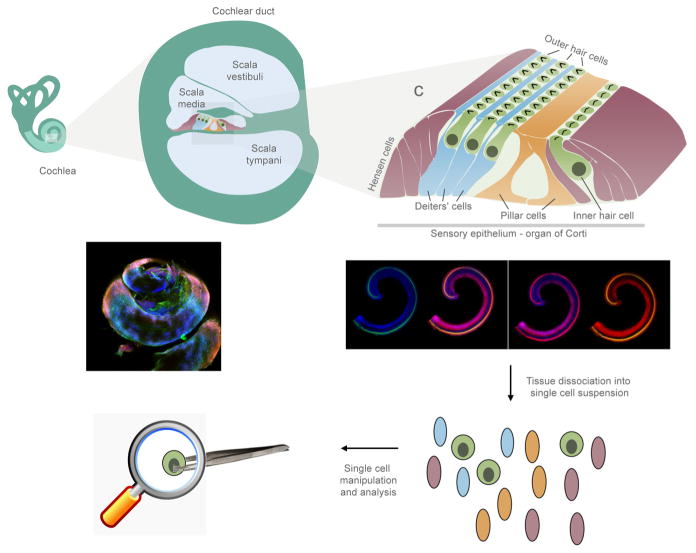
Schematic illustration and immunofluorescence of the inner ear, demonstrating the cells that may be analyzed on a single cell level The sensory epithelium of the cochlea is composed of one row of inner hair cells and three rows of outer hair cells, the sensory cells of the inner ear, and supporting cells. Adapted from (Dror and Avraham, 2009). Whole-mount cochlear preparations derived from newborn mice labeled with antibodies. Myosin VI labels the cytoplasm of the inner and outer hair cells, NF200 labels the neurofilaments and phalloidin labels the actin-filled stereocilia (for experimental details, see Elkan-Miller *et al.*, 2011).

The vertebrate inner ear provides sensory information about sound, motion, equilibrium and spatial orientation. These critical capabilities are mediated by sensory epithelial organs found in the inner ear (Fig. 1). Balance and perception of vertical location is mediated by five sensory patches in the vestibular portion of the inner ear, while sound is received in the cochlear portion of the inner ear by a single sensory patch named the organ of Corti. Both types of sensory patches present a very small, but highly complex tissue, composed of a mixed population of cells, developed and arranged in strict precision to allow the entire organ to function properly. The loss of either results in a malfunctioning of the sensory organs and compromises the relevant sense. Of these specialized cell types, the mechanosensory hair cell is responsible for translating small movements or changes in pressure waves to an electrical signal sent to the brain and perceived as sound, or either angular or linear acceleration (Groves and Fekete, 2012).

The entire inner ear originates out of one of the cranial placodes, giving rise to most of the craniofacial sensory organs. The development of each placode is orchestrated by temporal and spatial gradually occurring signals, leading to placode identity and morphogenesis, and maintaining a balance between essential progenitor cells embedded in the tissue, lineage restriction and final differentiation of cell types (Lleras-Forero and Streit, 2012; Schlosser, 2010). The otic placode develops into a multi-sensory inner ear. Once a bifurcation event distinguishes the otic placode from the rest of the pre-placodal domain, a hollow sphere morphology is formed, and from that point onwards the otocyst develops into a functional inner ear. This process is dependent on the location along three axes of the sphere, finally establishing sensory and non-sensory structures in their strictly defined locations and relative positioning to one another (Kelley, 2006). Although major efforts have been made in characterizing the molecular mechanisms and regulatory genetic networks governing the development and function of the inner ear sensory organs, our knowledge is just coming of age (Kelley, 2006b; Scheffer *et al.*, 2015; Shen *et al.*, 2015). In recent years, our understanding of genetic modules and key regulators of mechanosensory organ formation has increased through the use of high-throughput next-generation sequencing (NGS) and ultra-sensitive and low-input methodologies, together with bioinformatic computational advances.

As with other organogenesis processes in the body of a multicellular organism, the development of the inner ear is governed by a complex pattern of gene expression and non-coding regulatory elements. The development of the inner ear described above, with an emphasis on the complexity of functional inner ear sensory epithelia, has a very strict temporal and spatial regulation. As a result, the inner ear sensory organs are one of the most fascinating cellular structures among vertebrates amenable for single cell transcriptomic and epigenomic studies.

**Fig. 2:**
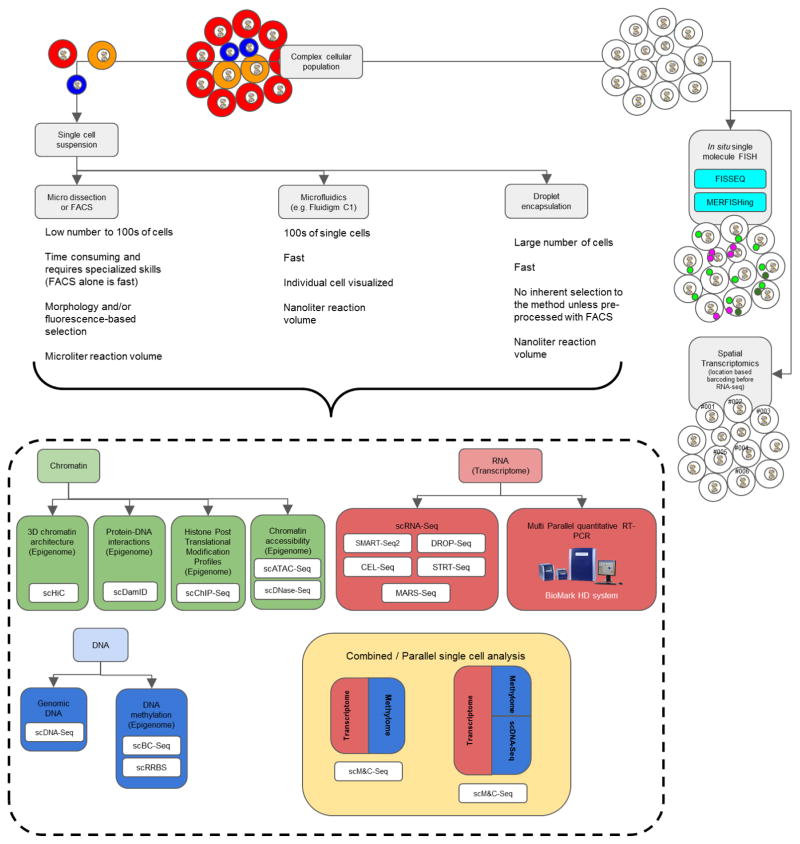
Methods for single cell analysis includes separation of single cells and subsequent processing A complex cellular population may be separated into single cells for transcriptomics and epigenomics by microdissection or FACS, microfluidics or droplet encapsulation, as described in more detail in the text. These same cells may be viewed from a spatial perspective by FISH or spatial transcriptomics, which may then be combined to achieve coupling of the single cell with spatial orientation. Adapted from (Clark *et al.*, 2016; Kolodziejczyk *et al.*, 2015).

The next phase in the world of molecular genetics, after the boost in high-throughput NGS, has been the rise of single cell analysis. Although NGS has revolutionized the fields of genetic and epigenetic research, each analysis required a pool of many cells, yielding a result representing the average of a cell population (Fig. 2). Today, many high-throughput applications are also available to the general research community at the single cell level (Cusanovich *et al.*, 2015; Jin *et al.*, 2015; Klein *et al.*, 2015; Macosko *et al.*, 2015; Nagano *et al.*, 2013; Rotem *et al.*, 2015; Saliba *et al.*, 2014; Smallwood and Ren, 2013). The analysis of single cell genomic and epigenomic features of the inner ear has the potential to change our entire perspective of its development known to date. Mapping the cell-to-cell variability and sorting out rare cellular populations and sub-populations will reveal novel cell lineage substructures. High resolution cell-to-cell variability advances our understanding of external and internal cellular processes and their effects on both transcriptome, epigenome and the phenotype, all of which were not possible prior to the resolution enabled today by various high-throughput single cell applications. We will attempt to summarize the state-of-the-art research in single cell analysis, provide a summary of the work performed to date in the inner ear field, and present further single cell analysis methodologies to be adopted for inner ear research.

## Single cell analysis

Single cell analysis, at the level of epigenetic regulation, transcriptome or protein translation dynamics, has led to a paradigm shift in the definition of regulatory and genetic plans of each cell type. Specifically, how a unique and highly specialized cell type is defined is being addressed anew. Using single cell analysis, scientists have begun to reformat cellular lineages by mapping cell-to-cell variation at all levels of regulation and gene expression at a resolution only dreamt of just a few years ago (Burns *et al.*, 2015; Ealy *et al.*, 2016; Schultze and Beyer, 2016; Zeisel *et al.*, 2015). Using the whole organ or even a selected cell population, sorted by a single marker to analyze the cell type-specific genetic regulatory network, yields an average transcriptomic and epigenetic profile that misses out on a subpopulation of cells. Transcriptional data derived from these critical cells is often lost as a result of averaging (Fig. 2). Single cell analysis of these same cellular populations, classically defined as a uniform cell type, revealed the cell-to-cell heterogeneity that could not be resolved as technical variation in all examined profiles. As single cell analysis is rapidly becoming embedded in various research niches in molecular biology, it is becoming clear that the cell-to-cell variations hold the key for discovery of new intermediate developmental stages or tissue-embedded progenitor cells that have not been previously characterized. The uniform definition of cell types is thus being replaced with a variable repertoire by redefining sub-populations and earlier developmental progenitor cells.

One such example of a complex tissue with a wide spectrum of cells is the brain. The brain is comprised of an extremely diverse population of specialized cell types responsible for, but not limited to, memory, sensorimotor functionality and interpersonal behavior. Annotating different cellular classes and subclasses is crucial to understand how specialized cell populations and cellular niches in a large organ structure perform various tasks. Single cell analysis has facilitated the highest resolution possible, enabling the focus on cell-to-cell variation even in a complex tissue such as the brain.

Cell type classification was performed in the mouse cerebral cortex by single cell RNA sequencing (scRNA-Seq) of 3005 cells from two defined regions of the brain (Zeisel *et al.*, 2015) (Fig. 2). The scRNA-Seq output was coupled with the physical cell information using a BiClustering strategy, which led to the division of the transcriptomes into nine major classes of the somatosensory S1 cortex, each correlating to specific markers known to play a role in cell type function. The resolution of information and intra-class variability allowed the investigators to repeat the BiClustering analysis within each of the nine classes, further enabling classification of 47 subclasses distributed among the more classic cell clusters. This division was not random and was detected in multiple mice, which might hint of an evolutionary force in play to keep intra-cell class diversity. In addition, specific subclasses were associated with a set of individual transcription factors or whole genetic modules, providing both regulatory and molecular explanations about subclass cell type functional specialization.

In the realm of another widely explored and well characterized cellular population, bone marrow and differentiated red and white blood cells, single cell analysis has broken down boundaries of known cell types by elucidating distinct cell-to-cell variation associated with differences in cell type and subtype function. Single cell transcriptomics, rather than biased cell surface marker methodology, has expanded the resolution of the myeloid differentiation tree to reveal new subgroups of progenitors (Paul *et al.*, 2015). Furthermore, the single cell transcriptome profiles indicate transcriptional priming towards a target cell fate. The key to their work was the combination of Massively Parallel RNA-Seq (MARS-Seq) and information derived from different methods such as fluorescence-activated cell sorting (FACS) on the cell surface markers of the analyzed cells, histone profiles, functional analysis and perturbation experiments (Fig. 2). The investigative depth of the single cell analysis performed allowed them to recapitulate a cluster-specific gene-regulatory network (GRN). The high resolution transcriptome and formation of cluster-specific GRNs revealed that the multiple primed cell state, traditionally thought to explain early lineage bifurcation events (Miyamoto *et al.*, 2002), are either extraordinarily rare or exist in a highly transient manner (Paul *et al.*, 2015).

## The inner ear: towards the single cell

The inner ear sensory epithelia, containing mechanosensory hair cells, enable two very distinct functions, namely hearing and balance (Fig. 1). Both the cochlear and vestibular sensory organs rely on the hair cells to execute these functions. Aside from the functional variability and crude transcriptome variance between cochlear and vestibular hair cells (Elkan-Miller *et al.*, 2011; Rudnicki *et al.*, 2014; Scheffer *et al.*, 2015; Shen *et al.*, 2015), there is a lack of high-resolution differential data on the finite transcriptome and regulatory differences between the specialized cell types of the inner ear sensory organs. Moreover, we lack information on the intra-cochlear or intra-vestibular cellular variability explaining, for example, perception of gradual frequencies of sound waves along the organ of Corti and the different tasks of the vestibular organs, respectively. The recent years has seen the beginning of single cell analysis in these sensory organs.

The early developmental stages of the inner ear sensory organ were recently addressed using a single cell analysis technique, allowing the researchers to distinguish primary tissue that later became the sensory organ of the inner ear from other cranial placode sensory organs and other neuroectoderm tissues (Durruthy-Durruthy *et al.*, 2014; Durruthy-Durruthy and Heller, 2015). They used highly parallel quantitative real-time PCR (qRT-PCR) of 382 single cells to reconstruct the developing mouse otocyst and early neuroblast lineages. They focused on 96 genes (92 otic markers and four control genes), which was the basis for multivariant analysis and clustering of cells according to common gene expression profiles. A reporter transgene mouse was used, Pax2^Cre/-^;Gt(ROSA26)Sor^tdTomato,mEGFP^, to initially separate between Pax2-driven EGFP expressing cells to obtain a purified otocyst and delaminating neuroblast cells versus non-otic surrounding cells in the embryonic day (E)10.5 developing mouse embryo (Hartman *et al.*, 2015). The use of two independent unbiased grouping algorithms resulted in two main groups, otocysts and neuroblasts, which in turn were subdivided into six clusters with distinct transcriptional profiles achieved by the BiClustering method (Durruthy-Durruthy and Heller, 2015). Although the investigators very adequately were able to show the clustering of distinct expression profiles of the neuroblast versus the otocyst, they did not provide any distinct relationship in the context of developmental processes. Despite lacking temporal or spatial information to understand the lineage tracing of the cells, the authors presented an idea of using a phase similarity network in order to transform the single time point data into a dynamic network, with the assumption that the spectrum of cellular clusters represents an earlier, progenitor cell type at one end, and at the other end, a more differentiated one. Careful analysis resulted in the separation of early and late neuroblast cells found in an intermediate state. Finally, they constructed a 3D sphere model for the otocyst, based on differential expression of genes associated with the three main body axes – dorsal/ventral, medial/lateral and posterior/anterior (Durruthy-Durruthy *et al.*, 2015). This 3D model, empowered by expression data, provided an opportunity for presenting signaling pathways and morphogen gradual influences on the developing otocyst.

To understand otic lineage key regulators, a direct approach was taken by analyzing otic sensory lineage populations from the microdissected E10.5 otic vesicle and post-natal day (P)3 cochlear and supporting cells (Hartman *et al.*, 2015). Although the RNA microarray approach resulted in a lower resolution, this work provided a strong basis for future experiments based on highly parallel single cell qRT-PCR or scRNA-Seq.

The conclusions and robustness of previously described tools (Durruthy-Durruthy *et al.*, 2014; Durruthy-Durruthy *et al.*, 2015; Durruthy-Durruthy and Heller, 2015; Hartman *et al.*, 2015; Ronaghi *et al.*, 2014) was used to explore various new aspects of the organ of Corti’s cellular population. A subset of 192 genes were examined from a series of 960 sorted single cells that represented the nine predefined cell types of the medial to lateral axis (Waldhaus *et al.*, 2015). Aside from enhancing the conclusions of previous works and elaborating the single cell transcriptomic dataset for the organ of Corti, they managed to present new insights about molecular mechanisms, leading the way to a deeper understanding of *in vivo* directed reprogramming, or regeneration, of cells in the organ of Corti, mostly by utilizing resident supporting cells and their transdifferentiation potential. The guiding concept was to track highly differentially expressed genes between inner and outer pillar cells of the apical region, given that inner pillar cells, as opposed to outer pillar cells, present with a regenerative feature (Cox *et al.*, 2014; Li *et al.*, 2015; Liu *et al.*, 2014; White *et al.*, 2006). This analysis revealed a primed cellular state in the inner pillar cells, presenting the canonical Wnt pathway with lower Notch effectors. Another key insight of their analysis was the single cell resolution to differentiate between hair cells on the apical-basal axis to underlying emerging tonotopy. They identified a subset of genes associated with hair cell maturation, which present a gradient in their expression between the analyzed apex, middle and base section of the organ of Corti.

## Coupling the single cell with spatial orientation

To date, single cell analysis research in the inner ear field has not integrated the differential expression of genes with high-resolution spatial orientation. There are currently an assortment of published techniques offering parallel or sequential analysis of both the transcriptome and the morphological and spatial characteristics of the cells, such as fluorescent *in situ* RNA sequencing (FISSEQ) (Lee *et al.*, 2014; Lee *et al.*, 2015), *in situ* sequencing for RNA analysis by sequential hybridization (Ke *et al.*, 2013; Lubeck *et al.*, 2014), and Multiplexed Error-Robust FISH (MERFISH) (Shalek and Satija, 2015) (Fig. 2). These techniques provide spatial information on every cell analyzed, while allowing for a single cell view of hundreds of RNA molecules to a single molecule level. These techniques still lack the high-throughput aspects of scRNA-Seq, and instead, work with a predesigned set of gene probes.

This issue was addressed in a recent work describing “spatial transcriptomics”, which provides spatial information by tagging cell-specific transcriptomics from tissue sections (Ståhl *et al.*, 2016). This method is based on introducing molecular barcodes into the synthesized cDNA captured over a dense barcoded oligo-dT array, followed by detaching the tissue, but not before capturing an image of the hematoxylin and eosin (H&E)-stained tissue structure over the array. The barcodes are used to carry the information regarding the cells’ tissue localization, tagging the specific transcriptome dataset and allowing the analysis of expression in the context of the intact tissue structure. Captured RNA goes through T7-based *in vitro* transcription (IVT) for pre-amplification, which later is used as input for RNA-Seq. The authors were able to demonstrate that 95% of the genes found in the bulk cell suspension-based RNA-Seq were found annotated by the “spatial transcriptome” methodology, spanning even low expressing mRNAs. This method was shown to be relevant for human breast cancer and mouse olfactory bulb tissue sections, both exhibiting distinct morphological structures. As a result, we predict that this method will offer a solution for coupling the single cell transcriptome and its spatial context for the organ of Corti.

Other solutions may be found in the computational realm, combining previously derived data such as *in situ* hybridization with newly produced unbiased scRNA-Seq, as demonstrated in the brain of marine segmented worms (Achim *et al.*, 2015).

## Re-thinking tissue developmental lineages

The paradigm shift in the question “what is a cell type?” is not the only one the single cell revolution is responsible for. Unraveling cellular heterogeneity on a single cell level also resulted in re-thinking the tissue formation and cell differentiation processes. Cellular lineage studies and genetic programming, explaining cell type commitment, have been studied extensively throughout the years in heart (Bruneau, 2013), liver (Si-Tayeb *et al.*, 2010), lung epithelium (Roos *et al.*, 2012) and the hematopoietic system (Seita and Weissman, 2010), resulting in very detailed differentiation schemes and cellular lineage trees. The resulting genetic programs and fate maps of cell lineages have advanced the fields of in vitro-directed differentiation of embryonic stem cells (ESC) and Induced pluripotent stem cells (iPSC) to enable regenerative medicine (Atala, 2015; Azadeh *et al.*, 2016; Costa *et al.*, 2015; Jessop *et al.*, 2016; Sasai, 2013; Wobma and Vunjak-Novakovic, 2016; Wu and Hochedlinger, 2011).

The next phase of dissecting cellular lineages has come from high-resolution “omics” data, provided from Multiplexed Parallel qRT-PCR, scRNA-Seq and other types of single cell analysis. More samples for each time point, smaller interval time points for testing differentiation processes, coupled with the single cell perspective, has resulted in higher resolution data and a finer mapping of the developmental processes comprising the tissue lineage tree and cell type commitment drivers and regulators. The critical question today is not necessarily the single bifurcation-type chain of events from ESC to committed early progenitors, but rather the control of gradual, almost continual progression of the multipotent cells through various progenitors along their distinct lineages until the formation of the fully functional complex tissue. The lineage trees produced using the high-resolution single cell data are broader, with more branching out from the early trunk to the treetop.

One of the major advantages of the higher resolution single cell data output is the release from the need for a priori knowledge of stage-specific cell characteristics. As a result, asynchronous cell populations can be separated based on the cluster analysis of single transcriptomes, without the bias of pre-selecting the cell with known cell surface markers. An example is presented in a single cell transcriptomic analysis on 198 cells, representing four time points along the formation of the distal lung epithelium (Treutlein *et al.*, 2014). The initial sampling of cells and clustering was not based on a priori knowledge of cell type specific markers. Their approach rectified the previously known classical model for distal lung epithelium development, with the higher resolution data of single cells resulting in the discovery of previously unknown developmental states and cell types embedded into the classical model. Their database allowed them to go one step further and analyze the significance of genes’ co-expression within a cluster and annotate the function of any specific cell cluster/cell type, based on its genetic module expression profile. Closing the circle on the traditional mode of cell type specific exploration, scRNA-Seq yielded many new cell type specific markers, offering a refinement of separation between closely related cell types, and provided the basis for a more precise work to identify intermediate states in the hierarchy by massively multiplexed single cell qRT-PCR.

Single cell analysis has guided much of the recent work in re-charting the blueprint of embryogenesis in mice and humans, on the levels of both the transcriptome and epigenome (Blakeley *et al.*, 2015; Klein *et al.*, 2015; Tang *et al.*, 2010). The single cell analysis of stem cells and the genomic dynamic during bifurcation events opened a window into the role of heterogeneity of extracellular stimuli response of stem cells and later progenitor line as a major part of the multicellular organism embryogenesis and organogenesis (Junker and van Oudenaarden, 2015; Klein *et al.*, 2015; Semrau *et al.*, 2015; Semrau and van Oudenaarden, 2015).

The efforts to define pluripotent cell differentiation into a functional organ of Corti have not yet utilized unbiased RNA-Seq. The developmental trajectory of the human otic lineage was delineated using an *in vitro* model in order to refine stem cell guidance protocols (Fritzsch *et al.*, 2015; Kelley, 2007; Koehler *et al.*, 2013; Koehler and Hashino, 2014; Li *et al.*, 2016). These efforts are part of a cellular and regenerative therapy approach for hearing impairment.

A set of 90 pre-selected genes, measured by multiparallel qRT-PCR, facilitated the discrimination between early Non-Neuronal Ectoderm (NNE), Pre-Placodal Ectoderm (PPE) and the anterior and posterior cranial placodes, the latter yielding the otic placode (Ealy *et al.*, 2016). Using the Monocle algorithm (Trapnell *et al.*, 2014), the single cell transcription profiles were ordered on a pseudo-temporal trajectory, mimicking the differentiation process in order to explain the dynamic changes during *in vitro* differentiation. The same was done using both the human ESCs (hESCs) H9 cell line and an iPSC line. The data derived from these cells was compared to the relevant E10.5 naïve otocysts from a mouse model. A comparison of 23 genes showed that both cell lines, after 12 days of *in vitro* differentiation, were closely related to the naïve mouse otocyst. The authors propose that the results demonstrated the value of using single-cell gene expression analysis to provide the key variables that might be introduced into cell cultures to obtain a desired cell lineage.

Although highly innovative, the *in vitro* micro-environment of cultured cells likely lack inter-cellular signaling and morphogens that drive the formation of the otic lineage. To overcome this issue, subsequent work has been performed directly on neonatal inner ears. A P1 mouse expressing *Lfng-*driven GFP and *Gfi1 Cre*-driven dTomato fluorescent markers was used to separate utricle and cochlear supporting cells and hair cells from the rest of the inner ear tissue (Burns *et al.*, 2015). scRNA-Seq of the marker-based separated cells and subsequent clustering and analysis of the transcriptomes on a pseudo temporal trajectory led to the identification of a novel pro-sensory domain at the edge of the cochlear sensory epithelium, where cells are still in a transitional phase at P1, poised for either sensory or non-sensory fate. The conventional notion of the development of the sensory epithelium and fate mapping of sensory hair cells versus non-sensory supporting cells was previously based on bulk RNA-Seq transcriptomes and missed this unique small, but very important, subpopulation. Another major change to the known inner ear lineage tree was the re-organization of the vestibular and cochlear branches compared with one another. The difference between sensory and non-sensory cell types was now shown to be much larger than the difference in the genetic program of sensory hair cells of the utricle and cochlea, bringing a portion of the vestibular and cochlear branches closer together transcriptionally than previously considered.

## Re-inventing time to explain the inner ear developmental trajectory

The high-resolution transcriptome output of single cell analysis, particularly scRNA-Seq, enables the analysis of cells from the developing tissue as if it was a snapshot of a linear, ongoing process, which is inherently asynchronous. If we define the developing tissue’s various cells, of all characteristics, as intermediate waypoints on a trajectory towards a stable and finally differentiated cell type, than each snapshot of cells we took as input of scRNA-Seq, include transcriptomic profiles from various points on the predefined trajectory. This methodology is named “pseudotemporal” ordering, whereby the transcriptomes of single cells are ordered along a synthetic temporal axis aligned with the biological process trajectory vector (Reid and Wernisch, 2016). The biological process might be the differentiation and/or branching of multiple cell fates stemming from a unique progenitor cell, or the gradual response for an extracellular stimulus or morphogen. The pseudo-time method was already in wide use when microarray usage was prevalent (Magwene *et al.*, 2003), Coupling the pseudo-time method with NGS examines the entire transcriptome instead of a subset of genes, allowing for higher resolution and sensitivity for mapping complex processes.

The issue of asynchronicity of the genetic plan and transcriptomes of similar cellular states became more relevant in the age of multi-parallel qRT-PCR and scRNA-Seq, as the amount of data and resolution increased. There are several freely available *in silico* toolkits developed to enable technologies to adapt to high-resolution data, such as Monocle (Trapnell *et al.*, 2014). This tool was used in the inner ear field to define the trajectory as the gradual differentiation process from pluripotent hESC towards the otic placode (Ealy *et al.*, 2016). Utilizing pseudotemporal analysis enables handling of cell-to-cell heterogeneity and translation of this information to developmental dynamics of organs, tissue and adult stem cell populations. Both are the major challenges of classical experimental designs of an averaged population of cells, as adult stem cells are a minority inside complex and heterogenic tissue. Another widely used toolkit has been developed, named ‘Waterfall’, for use on single-cell transcriptomes of the adult hippocampal quiescent neural stem cells (qNSC) to elucidate the key factors underlying the qNSC activation and eventually neurogenesis (Shin *et al.*, 2015). Tools for Single Cell Analysis (TSCAN), another more advanced tool available for pseudo-time reconstruction, took several lessons learned from previously available tools described above, and tried to make the process more accessible to user’s by first transferring to a GUI-based working environment instead of a command line-based one, reducing the cellular lineage tree complexity by various means (Ji and Ji, 2016).

Given the high heterogeneity of single cells, previously considered a uniform population, it is not feasible to set the number of cells needed to be sampled and analyzed in order to fully characterize all of the tissue’s cellular populations and sub-population at a reliable resolution. Sampling of an increased number of cells leading to higher resolution of lineage decomposition is possible; however, sequencing depth for each transcriptome is compromised with current technologies. If standard depth is kept with a larger sample number to cover as many of the cells of a given tissue, the costs become unreasonable. However, lowering the depth for each cell to as low as 50,000 reads may still enable clustering of unique cell types, for example, as was done for primary neural cells in order to discover new candidate biomarkers in the developing cortex (Pollen *et al.*, 2014). Novel methods allowing for the manipulation of single cells in nanoliter volume droplets such as inDrop (Klein *et al.*, 2015) and Drop-Seq (Macosko *et al.*, 2015) will likely increase the throughput and lower the cost, allowing more single transcriptomes representing more cells of solid tissues to be examined, without compromising the read depth.

Despite the progress described above, a true measure for evaluating and comparing the different tools and algorithms for pseudo-time reconstruction is still elusive. The single cell omics research community aims to provide a holistic pipeline to resolve various biological processes, from single cell omic analysis, which could be highly beneficial for studying inner ear sensory organ development towards understanding trans-differentiation and regeneration in the inner ear.

## The single transcriptome and the single epigenome

During the lifespan of the adult specialized cell, gene expression is regulated in part by epigenetic modifications such as DNA methylation, nucleosome occupancy, histone post translational modifications (PTMs), and chromatin structure, all of which have been in the spotlight in recent years and the driving force behind the formation of major research consortiums (Bernstein *et al.*, 2010; Kundaje *et al.*, 2015; Neph *et al.*, 2012; Xie *et al.*, 2013; Yue *et al.*, 2014). As for transcriptomics, the next leap of epigenomic research is in the realm of single cell analysis. It is expected that if the downstream level of RNA transcription is highly variable and dynamic on a cell-to-cell comparison, the level of regulation of the transcription process should exhibit a level of variability as well, allowing for fine tuning of transcriptomes. Studying epigenomics at a single cell level could reveal subtle dynamics, completely missed in bulk analysis (Macaulay and Voet, 2014). During the last two years, single cell epigenetic technologies have appeared such as single-cell DNA methylation (Farlik *et al.*, 2015; Guo *et al.*, 2013; Smallwood *et al.*, 2014), scChIP-Seq (Cusanovich *et al.*, 2015; Jin *et al.*, 2015; Klein *et al.*, 2015; Macosko *et al.*, 2015; Nagano *et al.*, 2013; Rotem *et al.*, 2015; Saliba *et al.*, 2014; Smallwood and Ren, 2013), and nucleosome accessibility assays such as scDNasI-Seq (Jin *et al.*, 2015) or scATAC-Seq (Buenrostro *et al.*, 2015). Mapping long distance chromatin conformation by resolving the cis- and trans-regulatory interactions that take part in the genome (Libbrecht *et al.*, 2015; Rao *et al.*, 2014) also moved toward single cell analysis with single cell HiC (Nagano *et al.*, 2013; Nagano *et al.*, 2015). The strength of these techniques lies in the multiparameter analysis of a single cell, directly correlating subtle changes in the regulatory epigenome and the resultant transcriptome for a specialized cellular phenotype.

This effort is still an emerging one, demonstrated by pioneering work connecting cell-to-cell variability of both the gene expression profile and the coupled epigenome by sequencing the transcriptome and methylome of the same single cell (Angermueller *et al.*, 2016; Hou *et al.*, 2016). This work led to the conclusion that epigenetic heterogeneity is the key switch component in the fluctuating pluripotency of serum ESCs. Based on this work, it is logical to conclude that other tissue types, such as the highly complex inner ear sensory epithelium, with features of differentiation, development and maturation to achieve function, is governed by cell-to-cell variability of the transcriptome, epigenome and its phenotype.

## Conclusions

**Fig. 3:**
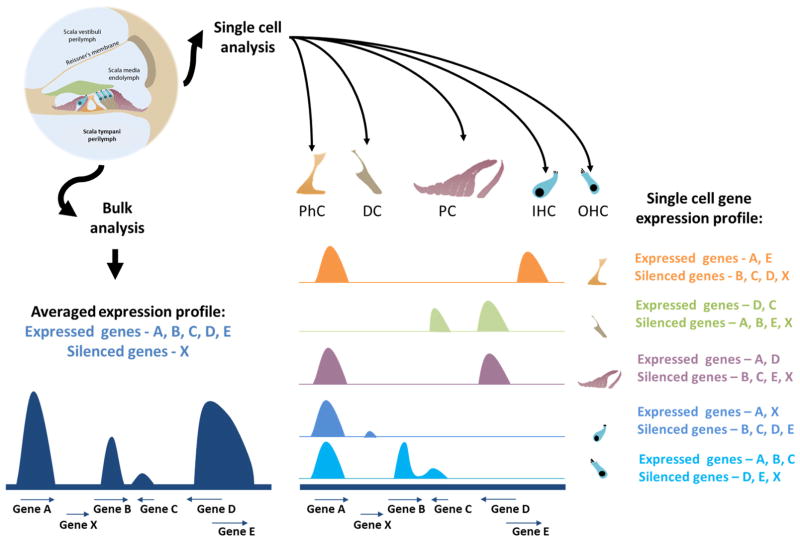
The current and future state of single cell analysis in the mammalian inner ear scRNA-Seq has been used for averaged gene expression profiles in inner ear research, providing data regarding gene expression and gene silencing for a subset of genes. As separation techniques improve for the organ of Corti and other portions of the inner ear, a more robust separation of the sensory inner (IHC) and outer hair cells (OHC) and non-sensory supporting cells, including pillar cells (PC), Deiters’ cells (DC) and inner phalangeal cells (PhC), may be made. The cell-specific genetic features will define which genes are expressed and which are silenced during different stages of development, and increase our knowledge regarding cellular function and mechanisms.

Despite some progress in single cell analysis of cell-to-cell variation in the inner ear in recent years, major progress in the field will come from making a transition from a pre-selected group of genes to performing robust scRNA-Seq to map out the entire transcriptome. Future experimental designs should focus on two main points: scRNA-Seq and a priori knowledge on the cell that includes spatial localization in the tissue, cell surface markers and/or reporter gene models. Examining transcriptomes of single cells of the inner ear sensory organs, instead of averaged gene expression profiles, will reveal cell-specific genetic features important for specific cellular functions and driving forces for organogenesis (Fig. 3). Similar strategies, as presented in this review on non-inner ear cells, can be pursued in virtually any inner ear cell type, bridging cell-to-cell transcriptome variability to function. Moreover, these strategies may be used to fully characterize the set of developing cell types comprising the inner ear sensory organs’ lineage hierarchy. Understanding the finite differences and dynamic changes among the cells of the inner ear present the greatest promise for regenerative therapy, as it will enable reconstruction of the cell lineage roadmap of the auditory and vestibular systems.

## Abbreviations used in this paper

E: embryonic day
ESC: embryonic stem cell
FACS: fluorescence-activated cell sorting
FISSEQ: fluorescent *in situ* RNA sequencing
GRN: gene-regulatory network
iPSC: induced pluripotent stem cell
MARS-Seq: massively parallel RNA-seq
MERFISH: multiplexed error-robust FISH
NGS: next-generation sequencing
P: postnatal day
qNSC: quiescent neural stem cell
qRT-PCR: quantitative real time PCR
scRNA-seq: single cell RNA sequencing
TSCAN: tools for single cell analysis
